# A Virtual Instrument System for Determining Sugar Degree of Honey

**DOI:** 10.1155/2015/534795

**Published:** 2015-10-04

**Authors:** Qijun Wu, Xun Gong

**Affiliations:** ^1^School of Chemical Engineering, Guizhou Institute of Technology, Guiyang 550003, China; ^2^School of Chemical Engineering, Guizhou University of Engineering Science, Bijie 551700, China; ^3^School of Light Industry, Guizhou Institute of Technology, Guiyang 550003, China

## Abstract

This study established a LabVIEW-based virtual instrument system to measure optical activity through the communication of conventional optical instrument with computer via RS232 port. This system realized the functions for automatic acquisition, real-time display, data processing, results playback, and so forth. Therefore, it improved accuracy of the measurement results by avoiding the artificial operation, cumbersome data processing, and the artificial error in optical activity measurement. The system was applied to the analysis of the batch inspection on the sugar degree of honey. The results obtained were satisfying. Moreover, it showed advantages such as friendly man-machine dialogue, simple operation, and easily expanded functions.

## 1. Introduction

LabVIEW is a G language-based virtual instrument (VI) software development tool compiled by National Instruments Corporation. It is mainly used for data collection, analysis, measurement, instrumentation, process monitoring, and so forth. LabVIEW integrates a large number of interface templates for graphic generation. Moreover, it owns abundant numerical analysis controls, advanced acquisition controls, signal analysis controls, perfect simulation debugging tools, and a variety of hardware device driving functions, including RS-232, GPIB, VXI, VISA, and DAQ. It can be used to rapidly develop the virtual systems of data acquisition and analysis control in short development cycle. LabVIEW has been widely used in many fields worldwide including aviation, aerospace, communication, automobile, semiconductor, and biomedicine [1, 2].

Polarimeter is an optical instrument for measuring the optical activity of material. It is commonly used to measure the optical activity of optically active substance. Through measuring the optical activity of the sample, it has access to determine the concentration and purity of a substance and analyze the configuration of organic matters. The optical rotation of a honey depends on the concentrations of the various sugars present in the honey, and this can be used for differentiation between nectar honeys (laevorotatory; negative values of optical rotation) and honeydew honeys (dextrorotatory; positive values of optical rotation). The rotation properties are very important for honey characterisation, in order to distinguish and assess the declared botanical origin of the honey [3, 4].

WZZ-2B automatic polarimeter is a test instrument commonly used in China currently. The optical activity is measured based on the photoelectric automatic balance principle and digitally displayed on a light-emitting diode (LED). However, this instrument does not have the functions of data acquisition and intelligent data processing. In the actual application, the user needs to further analyze and process the data measured to obtain the final results. According to the characteristics of automatic polarimeter, this study developed a LabVIEW-based VI system for measuring the optical activity of materials by connecting the instrument with the computer through the serial port. This system realized the automatic acquisition, processing, and result generation and intelligently completed the test task.

## 2. The Hardware Centrifugation of the System

**Figure 1:**
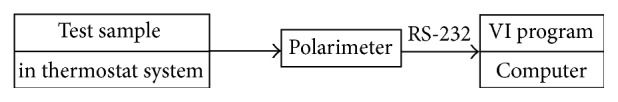
The structure diagram of the system.

The VI system is composed of computer, automatic polarimeter, CH1015 super thermostat bath, and LabVIEW-based program. The polarimeter tube is refitted to provide constant temperature for the tested sample. A polarized light signal after the sample tube is detected by PhotoMultiplier Tube (PMT) and converted into electronic signals, which could be transported into computer through serial port (RS-232). The structural diagram of the VI system is shown as in Figure 1.

## 3. The Programming of the VI System

The VI program system is constituted by some SubVIs including data acquisition and sugar content analysis, viewing recorded data and exiting the program [5–7]. It mainly used to complete the real-time data display, storage, data processing, and result playback tasks.

### 3.1. Programming for Data Acquisition

**Figure 2:**
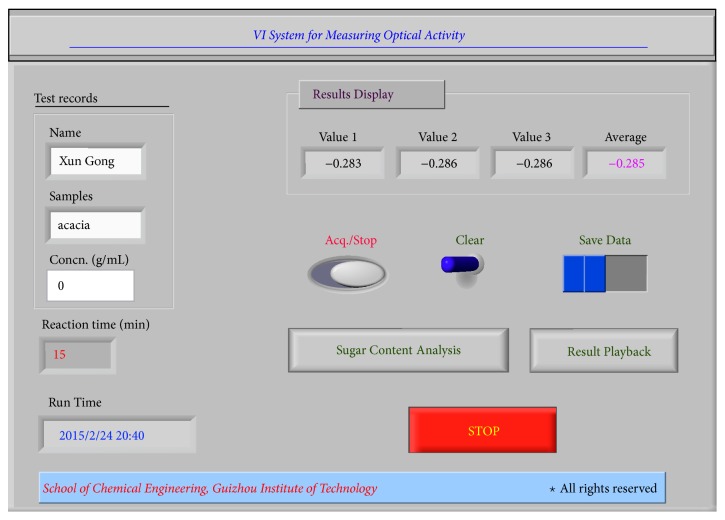
The main operation panel of the VI.

Figure 2 shows the main operation panel of the VI system. The right-hand part of this figure is used to display experimental data and choose the test tasks in real time, while the left-hand part is used to record the field information, such as experimental personnel, experimental samples, and reaction time. By clicking the button of “sugar content analysis,” researchers enter into the data processing SubVI to process the experimental data in real time. By clicking the button of “result playback,” it has access to playback the data results processed. By clicking the “stop” button, the operation of the VI system is terminated. The SubVIs in the programming call the Event Structure, which can realize the interactions between the operations of users on front panel and program execution.

**Figure 3:**
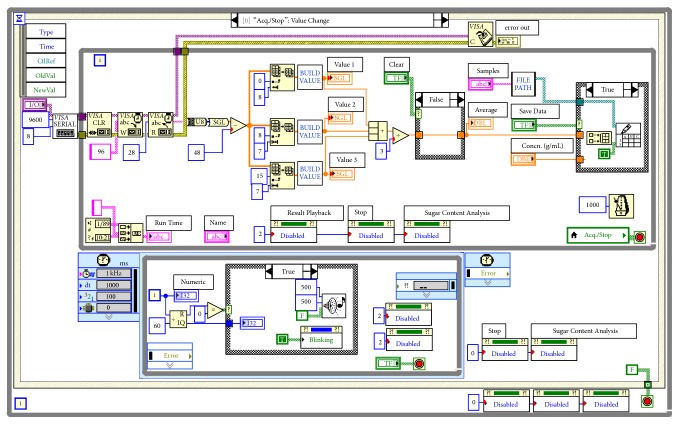
Program for the data acquisition.

Figure 3 is a program chart of the data acquisition of the system. The program of data acquisition is realized according to the data protocol sent by the serial port of the polarimeter [5, 7]. The initialization, writing, reading, and closing of the serial port, and so forth, were realized using the VISA nodes in LabVIEW. Before the communication of serial port, it is needed to set port parameters for the nodes of* VISA Configure Serial Port*. Using the* String To Byte Array* node, the data read by* VISA Read* node from the instrument cache are converted into integer array. Using nodes such as* Array Subset* and* Index Array*, the specified optical activity of each frame in the instrument can be read out.

The concurrent structure of* Timed Loop* and* While Loop* is used to accurately display the current measurement time.* Remainder* function is employed to report the accurate time at full minute and highlight the current acquisition time. Time-reporting and alarming sounds are realized through* Beep.vi*.

The Case Structure on the right part of the* While Loop* in Figure 3 is applied to optional save measuring data. File storage path is realized by “file path” subprogram. To avoid the file name repetition, a* List Folder* node is called. In case of indexing out the same file name, the file name is automatically added with a specific character string.* Write To Spreadsheet File.vi* function realizes the simultaneous saving of optical activity data and concentration data collected. Users can click the acquisition button on the operation panel of the VI to collect data. By clicking save data button, the data measured are saved.

Data acquisition of the program cannot be preceded with other events simultaneously. Other events are only allowed to be proceeding when data acquisition is ended. This function is realized by setting the attributes of the event buttons to avoid the conflict of the events called [7].

### 3.2. Programming for Sugar Content Analysis of Honey

After data acquisition, users can enter into the sugar content analysis operation panel. The program for the sugar degree analysis of honey mainly applied to measure the contents of glucose, fructose, and sucrose to detect honey quality. Such detection functions to determine whether or not the honey is adulterated or whether or not the honey is pure. The principle is indicated as follows [8, 9]: the total gross (*A*) of the reducing sugars (glucose and fructose) in the honey is firstly measured using potassium ferricyanide method. The contents of glucose, fructose, and sucrose are set as *x*, *y*, and *z*, respectively. Subsequently, according to the linear relation of optical activity and concentration, the optical activities of the honey solutions diluted by distilled water and hydrolyzed by sulfuric acid were measured. Honey after acid hydrolysis of sucrose can only complete hydrolysis into the same amount of glucose and fructose. In data processing, the measured data is multiplied by the dilution ratio to obtain the optical activity of original honey. The rotation [*α*]_*D*_^20^ for glucose, fructose, and sucrose are 52.5, −91.9, and 66.6. Therefore, there are *A* = *x* + *y*, *a*_1_ = 52.5*x* − 91.9*y* + 66.6*z*, and *a*_2_ = 52.5(*x* + *z*) − 91.9(*y* + *z*). By combining these equations, it has access to the sugar degrees of glucose, fructose, and sucrose, respectively.

**Figure 4:**
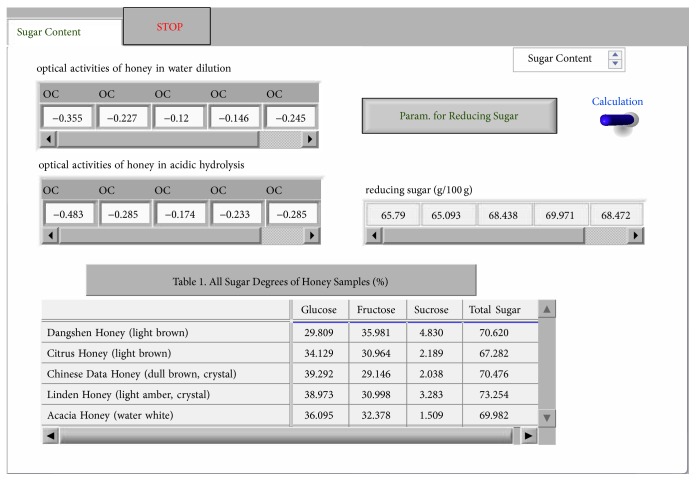
Panel for the sugar degree analysis.

Figure 4 shows the operation panel of the sugar degree analysis of honey. By clicking the button of “parameters for reducing sugar,” the SubVI panel for setting the parameters of reducing sugar is popped. After inputting the parameter of the reducing sugar and clicking the “returning” button, the reducing sugar of operation panel as in Figure 4 is returned. By inputting the optical activity of the honey solutions diluted by distilled water and that of the solution hydrolyzed by sulfuric acid and clicking “calculation,” the three sugar degrees and total sugar contents of the all honey samples were listed in the table, as shown by the lower part of Figure 4.

**Figure 5:**
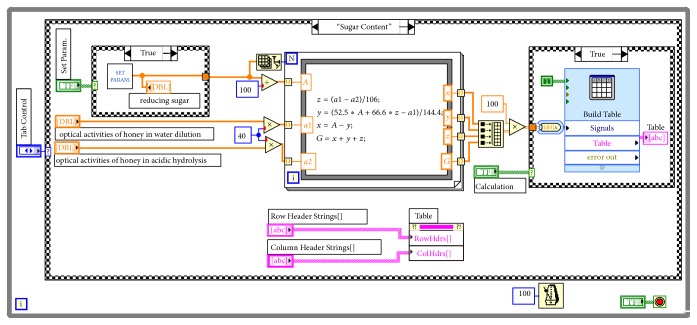
Program for the sugar degree analysis.

The* Formula Node* inside a* For Loop* in the programming is used to realize the calculation on the sugar degree of the honey.* Array Size* is used to index the sample number that connects to the count port of the* For Loop*; that is, the sugar degree of multiple different honey samples can be calculated separately. By calling* Express Table*, building the* Property Node of Express Table*, and selecting attributes for* Row Header Strings* and* Column Header Strings*, it is accessible to write the column and row heading for the listbox and record the honey sample and corresponding sugar degree in detail.  Figure 5 shows the program chart.

### 3.3. Programming for Datalog Playback

**Figure 6:**
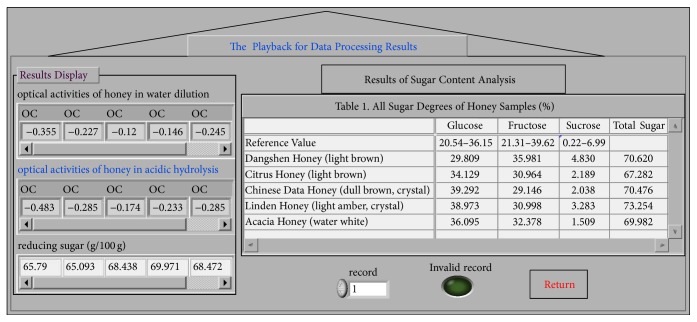
The playback panel for data results.

LabVIEW can record the current data of all the controls in the front panel of the program. Each time of data record gives rise to a record in the forms of the combinations of arbitrary types of data in the datalog file. Before the datalog on the front panel, it is required to select the menu command* Operate*→*Log at Completion* on the front panel of the data recorded. The reading of datalog file is realized by calling the data recorded VI, which is treated as the SubVI here. By right-clicking on the SubVI icons of this block diagram, a menu is popped. By selecting the* Enable Database Access* on the menu, it has access to the state of viewing the datalog. The data that needed to be read can be played back using the* Unbundle By Name* node.  Figure 6 shows the playback panel for data processing results. By selecting the number of the record that should be viewed, the datalog under this record number is displayed in real time. In case that there is no data under this record number, the Boolean LED is the highlight. By clicking “return,” the users can return to the main operation panel.

## 4. Results and Discussion

Owning to the high nutritional and medicinal value, honey has been widely applied to Chinese patent drugs and foods. Honey shows a complex composition, with more than 20 kinds of detectable ingredients. The main ingredients include fructose, glucose, and sucrose, accounting for about 70% of the honey in content. In addition, the three substances are optically active. Except for them, there are few other optically active substances in honey. Chinese honey standard stipulates that the reducing sugar content and sucrose content should take a proportion of above 65% and below 5%, respectively. For the exported honey, it is even required that the fructose should account for above 50% of the reducing sugar. The reason of these stipulations lies in that, on one hand, whether or not the honey is mixed with sucrose or starch substances is detectable. On the other hand, it can determine whether or not the honey contains the honey yielded by the bees fed by sucrose. Therefore, the natural characteristics and ingredients of pure honey can be reflected by measuring various sugars in the honey.

The analysis on the sugar degree of honey is preceded as follows. Firstly, the total contents (*A*) of the reducing sugar in all honey samples were measured using potassium ferricyanide method. Then, 25.00 g (in accurate weight) of citrus honey, linden honey, Chinese date honey, Dangshen Honey, and acacia honey (origin: Sichuan) was put into 100 mL volumetric flasks, respectively, and diluted using distilled water to constant volume. The honey solutions obtained were then discolored using activated carbon for 3 hours. After being filtered, we take two shares of 10 mL sample solution from each discolored solution and put these sample solutions in different 100 mL volumetric flasks. One of the solution samples was diluted with 10 mL 3 mol/L H_2_SO_4_, adding distilled water to the constant scale, and another was directly added into distilled water to the scale and placed overnight. In the following day, we measured the optical activities of the solutions diluted by acid and water at a constant temperature of 20°C. Afterwards, we input the reducing sugar parameter measured at the beginning and the optical activities of the honey solutions diluted and clicked “Yielding the Results.” The sugar degree data processing results of each honey sample were obtained, as shown in Figures 5 and 6. A reference value using China's standard sugar degree determination using the HPLC method is shown in Figure 6, glucose displays the highest content above reference value (like 39.292% and 38.973%), and it is easily crystallized; the contents of glucose and fructose are all higher than 65%, while sucrose contents are all less than 5%. The results obtained were consistent with the results obtained using China's standard sugar degree determination method. Therefore, it is proved that this VI system is applicable to the batch detection of a variety of honey samples.

## 5. Conclusions

The application results suggested that the LabVIEW-based optical activity measurement VI system conveniently solved the hardware problems of conventional chemical optical instrument such as the interface with computer and realized the automatic data acquisition and data storage functions. Moreover, using the rich function library of LabVIEW, users can compile different LabVIEW programs for this system to profoundly analyze and process the optical activity data measured and obtain corresponding measurement results in real time. It was applicable to the batch detection in the sugar degree analysis of honey. In addition, this system showed friendly man-machine interface and convenient operation.
